# Buffalo Academy of Medicine

**Published:** 1899-08

**Authors:** Thomas F. Dwyer


### BUFFALO ACADEMY OF MEDICINE.

Reported by THOMAS F. DWYER, M. D., Secretary.

THE quarterly meeting was held at the Academy parlors, Palace Arcade, Tuesday evening, June 27, 1899.

In the absence of the president, Dr. Matthew D. Mann, the meeting was called to order at 9 o'clock p. m., by vice-president Dr. Eli H. Long, chairman of the section of medicine.

The minutes of the last quarterly meeting and the special meeting of May 25, 1899, were read and approved. The council recommended the election of Drs. Francis E. Fronczak, F. J. Carr, Max Keiser, Irving P. Lyon, for resident membership and Dr. E. H. Ballou, Gardenville, N. Y., and Dr. Thomas J. McBlain, Niagara Falls, N. Y., for non-resident membership. They were separately ballotted for and elected Fellows of the Academy. The section of obstetrics and gynecology made provision to entertain the Academy with a paper by Dr. William P. Spratling, Craig Colony, Sonyea, N. Y., but the doctor at a late hour telegraphed the secretary his regrets that an important meeting of the board of managers of that institution prevented his being present.

Officers of the Buffalo Academy of Medicine, session 1899-1900: President, Matthew D. Mann; secretary, Thomas F. Dwyer; treasurer, Eugene A. Smith; trustees, Stephen Y. Howell, three years; Benjamin G. Long, two years; Marcel Hartwig, one year. Section of Medicine, chairman, Eli H. Long; secretary, J. C. Clemesha. Section of Surgery, chairman, J. Henry Dowd; secretary, Marshall Clinton. Section of obstetrics and gynecology, chairman, Henry D. Ingraham; secretary, Albert J. Colton. Section of pathology, chairman, Arthur G. Bennett; secretary, Frederick H. Millener. Council, the above officers and the president of the Academy of the previous year, Dr. Roswell Park.

The attendance at this meeting was 20 and it adjourned at 9.45 p. m.

